# Decrease in the expression of the type 1 PTH/PTHrP receptor (PTH1R) on chondrocytes in animals with osteoarthritis

**DOI:** 10.1186/1749-799X-5-28

**Published:** 2010-04-26

**Authors:** Christoph Becher, Thomas Szuwart, Philipp Ronstedt, Sven Ostermeier, Adrian Skwara, Susanne Fuchs-Winkelmann, Carsten O Tibesku

**Affiliations:** 1 Orthopaedic Department, Hannover Medical School, 30625 Hannover, Germany; 2 Institute of Anatomy, Westfalian Wilhelms University, 48149 Muenster, Germany; 3 Department of Orthopaedics and Rheumatology, Philipps University Marburg 35043 Marburg, Germany; 4 Sporthopaedicum Straubing, 94315 Straubing, Germany

## Abstract

**Background:**

To evaluate the expression of the type 1 PTH/PTHrP receptor (PTH1R) on chondrocytes from hyaline cartilage over the course of osteoarthritis (OA).

**Methods:**

In 12 NZW rabbits, the anterior cruciate ligament (ACL) was resected to create anterior instability of the knee. In 12 control rabbits, only a sham operation, without resection of the ACL, was performed. Four animals from each group were killed at 3, 6, and 12 weeks. After opening the knee joint, OA was macroscopically graded and hyaline cartilage of the load-bearing area was evaluated histologically according to the Mankin scale and by immunostaining for PTH1R.

**Results:**

There was a positive linear correlation between the time after surgery and the macroscopic and histologic OA scores. The scores in the control group were constant over the time course. Immunostaining showed significantly less expression of PTH1R in the experimental compared to the control group after 6 (P < 0.05) and 12 weeks (P < 0.01). In the experimental group, a negative linear correlation between PTH1R expression and macroscopic and histologic grades was found.

**Conclusions:**

The results show an in vivo decrease in the expression of PTH1R on chondrocytes over the time course of OA. Further studies are needed to evaluate whether new treatment approaches could evolve from this knowledge.

## Introduction

The type 1 PTH/PTHrP receptor (PTH1R) belongs to a family of G-protein-coupled receptors (GPCR) with seven membrane-spanning helixes [1]. The PTH1R acts as a common receptor for the Parathyroid hormone (PTH) and the Parathyroid hormone-related peptide (PTHrP) [1-3]. It is highly expressed in bone and kidney and mediates in these tissues the PTH-dependent regulation of mineral ion homeostasis [1]. The PTH1R is also highly expressed in the prehypertrophic chondrocytes of metaphyseal growth plates [4,5]. It was demonstrated that PTH has a stimulatory effect on proliferation of chondroprogenitor cells and inhibits collagen and matrix synthesis and thus regulates cartilage growth and chondrocytic apoptosis [6].

PTHrP is known as an important local factor for chondrogenesis by regulating chondrogenesis in a manner that attenuates chondrocyte hypertrophy [3,7]. By acting through a complex signaling network, PTHrP allows the bone to grow and elongate normally [1,7-9].

Osteoarthritis (OA) is one of the most common skeletal disorders characterized by cartilage degradation, osteophyte formation and thickening of the subchondral bone in joints. Since little is known about the underlying molecular mechanism, several experimental OA models have been developed for investigation [10]. Whereas the role of PTH1R in osteoarthritic chondrocytes remains largely unanswered, it was shown that as disease severity in OA progresses, PTH1R expression and protein levels are reduced in human subchondral osteoblasts [11]. It was suggested that OA osteoblasts may be responsible for the initiation of this disease since it was indicated that cocultures of OA osteoblasts with normal cartilage explants may initiate cartilage degradation [12].

Furthermore, a clear reduction was shown in PTH1R abundance in OA osteoblasts and that it was related to disease severity [11]. The expression of PTHrP in cartilage and OA was addressed in several studies. It was shown that PTHrP is released in high concentrations in OA [13] and is more abundant in knees of patients with OA than in normal human knee articular cartilage [14]. Furthermore, the expression of PTHrP in chondrocytes obtained from cartilage tissues from patients with osteoarthritis during hip replacement surgery was dependent on the degree of cartilage degeneration. Cartilage with moderate degeneration expressed more of this peptide than mildly or severely degraded cartilage specimens did [15]. However, all such studies dealt with late-stage OA because human specimens were available only at the time of surgery.

The present study was designed to evaluate the expression of the PTH1R on chondrocytes over the time course of experimentally induced secondary OA in an animal model as well as possible correlations between macroscopic and histologic findings in the degenerated hyaline cartilage.

## Materials and methods

Animal experiments were performed at the Department of Orthopedics at the University of Muenster. All procedures were performed with the permission of the local government's animal rights protection authorities and in accordance with the National Institute of Health guidelines for the use of laboratory animals (G49/2000). Twenty-eight female NZW rabbits were used in the study. All animals were fully grown, clinically healthy, and had a mean ± SD weight of 4,193.75 ± 298.84 gm at the time of surgery and a mean ± SD weight of 4,274.64 ± 462.49 gm at the time of death.

### Trial groups

The animals were divided into 3 groups. In 12 rabbits, the anterior cruciate ligament (ACL) was resected to create anterior instability of the knee (group 1). In 12 control rabbits, only a sham operation was performed, without resection of the ACL (group 2). The 4 animals of group 3 underwent no surgical treatment.

### Surgical procedure

All operations were performed under general anesthesia (intramuscular administration of ketamine and xylazine). All animals received an intramuscular injection of broad-spectrum antibiotics (Tardomyocel; Bayer, Leverkusen, Germany) as prophylaxis against infection. For postoperative pain medication, intramuscular metamizole (Novalgin; Aventis Pharmaceuticals, Bad Soden, Germany) was administered. Operations on both knee joints were performed under a single dose of anesthesia. The knee joints were opened using a medial paramedian skin incision of 3 cm in length and a medial parapatellar incision of the capsule. In animals of group 1, the ACL was resected using a tapering scalpel. The patella was dislocated laterally. In animals of group 2 (control group), the joint and skin were closed again with no further surgical treatment (sham operation). An intraoperative Lachman test was carried out to evaluate anterior instability. Before closure, the joints were rinsed thoroughly. Postoperatively, all animals were allowed to move freely in their cages (which measured 100 cm × 70 cm × 40 cm).

### Macroscopic evaluation

Four animals from each group were killed 3, 6, and 12 weeks after the operations. The 4 animals from group 3 (no surgical treatment) were killed after they reached maturity. The joints were evaluated macroscopically by using a self-developed grading system consisting of 4 different criteria: fibrillations and ulcerations of the hyaline cartilage, osteophyte formation, and joint effusion. Scores for fibrillations ranged from 0 for intact hyaline cartilage to 3 for marked fibrillations, scores for ulcerations ranged from 0 for normal to 2 for a large area of ulceration, scores for osteophyte formation ranged from 0 for no osteophytes to 3 for marked osteophyte formation, and scores for joint effusion ranged from 0 for no effusion to 3 for marked effusion. The total score ranged from 0 to 11, with 0 being a macroscopically intact knee joint and 11 being late-stage OA. After macroscopic grading, both distal femurs were immediately stored at ~80°C until further examination.

### Specimen preparation

Tissue samples (3-5-mm thick) were fixed in 4% buffered paraformaldehyde for 2 days. After decalcification with buffered EDTA (20% EDTA, pH 7.4), the samples were dehydrated and embedded in paraffin. Sections were cut at a thickness of 5 μm, mounted on poly-L-lysine-coated glass slides, deparaffinized in xylene, and washed 3 times with distilled water and then with Tris buffered saline (TBS; pH 7.5) for 2 minutes each (washing procedure). Some of the sections were stained with Safranin O or with hematoxylin and eosin (H&E) to evaluate histologic changes of the cartilage and bone tissue according to the Mankin scale [16]. The Mankin score included assessments of the structure, cellularity, Safranin stainability, and integrity of the tidemark. The scores for structure ranged from 0 for normal to 6 for complete disorganization of the cartilage, scores for cellularity ranged from 0 for normal to 3 for hypocellularity, scores for Safranin O stainability ranged from 0 for normal to 4 for no stainability, and scores for tidemark integrity ranged from 0 for an intact tidemark to 1 for blood vessels crossing the tidemark. The total score ranged from 0 to 14, where 0 = intact hyaline cartilage and 14 = late-stage OA.

### Immunohistochemical method for labeling PTH1R

Deparaffined sections were hydrated and incubated for 5 min in proteinase K (ready-to-use; Dako, Hamburg, Germany). After washing in TBS, the slides were treated for 10 min with 3% H_2_O_2 _and blocked for 30 min in 3% bovine serum. A monoclonal mouse anti-rabbit PTH1R was used as primary antibody (IgG1, clone VFF-18, Bender MedSystems, Vienna, Austria). The sections were incubated with the primary antibody in PBS + 1% bovine serum albumin (diluted 1:50) over night at 4°C. After washing once again sections were incubated for 30 min in the secondary antibody anti-mouse IgG (K4001; Dako) at room temperature. After washing in TBS, the reaction was developed with aminoethylcarbazole chromogen substrate (ready-to-use; Dako) for 30 minutes at room temperature. To demonstrate specificity of binding of mouse anti-rabbit PTH1R in control experiments, sections were incubated (a) without primary antibody or, instead of primary antibody, (b) mouse IgG1 with irrelevant specificity (Aspergillus niger glucose oxidase; Dako) was used at the same concentration as the primary antibody. In all controls tested the specificity no positive staining of the tissue slices was visible.

### Quantitative counting of cells

Pictures of histologic sections were taken with a CoolSnap-Pro Color camera (model A00J82025; Media Cybernetics, Silver Spring, MD). The cells were counted using Image-Pro Plus software for Windows, version 4.1 (Media Cybernetics). First, the total number of chondrocytes in an immunohistochemically stained section was determined. Afterward, the number of immunohistochemically stained cells was counted in the same section and measured in relation to the total number of chondrocytes. Zonal attribution of PTH1R positive chondrocytes was done with respect to the typical shape of chondrocytes at the different zones of articular cartilage according to Buckwalter et al. [17].

### Statistical analysis

Statistical analysis was performed using the Statistical Package for Social Sciences, release 11.0 (SPSS, Munich, Germany). Student's *t*-test was performed for comparison of scores in the control and experimental groups. Spearman's coefficient was calculated to determine correlations. *P *values less than 0.05 were considered significant.

## Results

Two animals developed postoperative hematomas,1 animal had a superficial wound infection and was treated with a single injection of antibiotics, and 1 animal was reoperated upon 2 days after surgery because of a wound gap. All 4 wounds subsequently healed with no sequelae. All other animals had no complications and, an average of 1 week after surgery, no joint effusion was found upon clinical evaluation.

When the animals were killed, 11 of the 12 rabbits in group 1 (ACL resection) did not have an ACL. One animal in group 1 had intact ACLs bilaterally and was taken out of the experimental group. All animals in groups 2 and 3 had an intact ACL at the time of death.

### Macroscopic grading of OA

A total of 54 joints were evaluated macroscopically. Signs of degeneration according to our self-developed grading system were found in all groups. The mean ± SD macroscopic grade of OA was 0.375 ± 0.518 in group 3 (control group). In group 2 (sham operation), it was 0.375 ± 0.744 after 3 weeks, 0.75 ± 0.886 after 6 weeks, and 0.625 ± 0.518 after 12 weeks. In group 1 (ACL resection), the mean ± SD macroscopic grade of OA was 6.625 ± 1.768 after 3 weeks, 6.5 ± 2.33 after 6 weeks, and 9.5 ± 1.049 after 12 weeks. Group 1 had statistically significantly higher values than group 2 at every time point *(P *< 0.01). The macroscopic grade of OA correlated positively with the number of weeks after the operation (r_s _= 0.519, *P *< 0.05).

### Histologic grading of OA

According to the Mankin score, group 1 (ACL resection) showed a highly significant increase in the grade of OA compared with the untreated control group and the sham-operated group (group 2). In group 3 (no treatment), the mean ± SD histologic grade of OA was 1.75 ± 1.581. In group 2 (sham operation), it was 3.25 ± 0.707 after 3 weeks, 1 ± 1.195 after 6 weeks, and 2.25 ± 1.165 after 12 weeks. In group 1 (ACL resection), the mean ± SD histologic grade of OA was 7.25 ± 3.615 after 3 weeks, 8 ± 2.878 after 6 weeks, and 9.83 ± 3.061 after 12 weeks. Group 1 had statistically significantly higher values than group 2 at every time point *(P *< 0.01). The average histologic grade of OA increased with the number of postoperative weeks. The macroscopic and histologic grades of OA were strongly correlated (r_s _= 0.770, *P *< 0.01).

### PTH1R staining by immunohistochemistry

**Figure 1:**
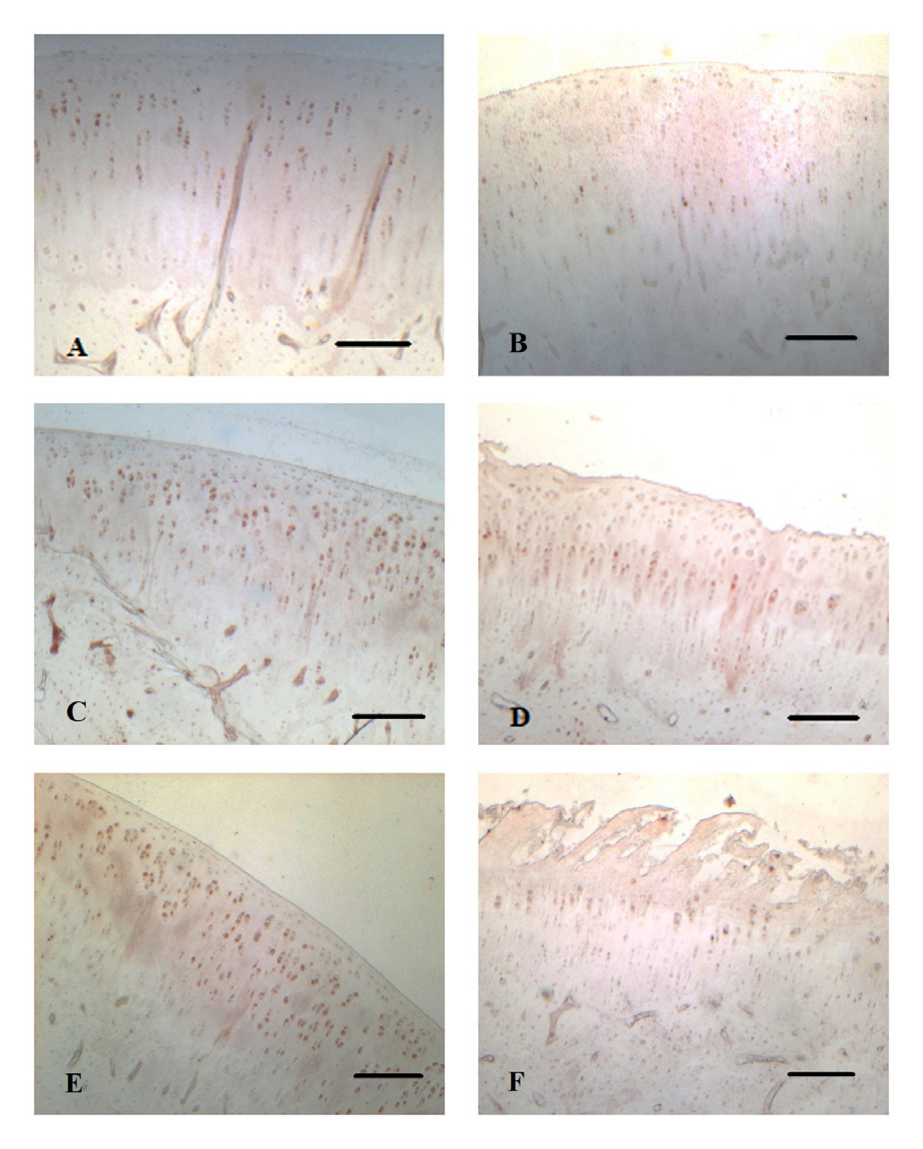
**Immunohistochemical staining of PTH1R**. **A**, PTH1R expression 3 weeks after the sham operation. **B**, PTH1R expression 3 weeks after resection of the anterior cruciate ligament (ACL). **C**, PTH1R expression 6 weeks after the sham operation. **D**, PTH1R expression 6 weeks after resection of the ACL. **E**, PTH1R expression 12 weeks after the sham operation. **F**, PTH1R expression 12 weeks after resection of the ACL. Magnification bar: 0,25 mm.

**Figure 2:**
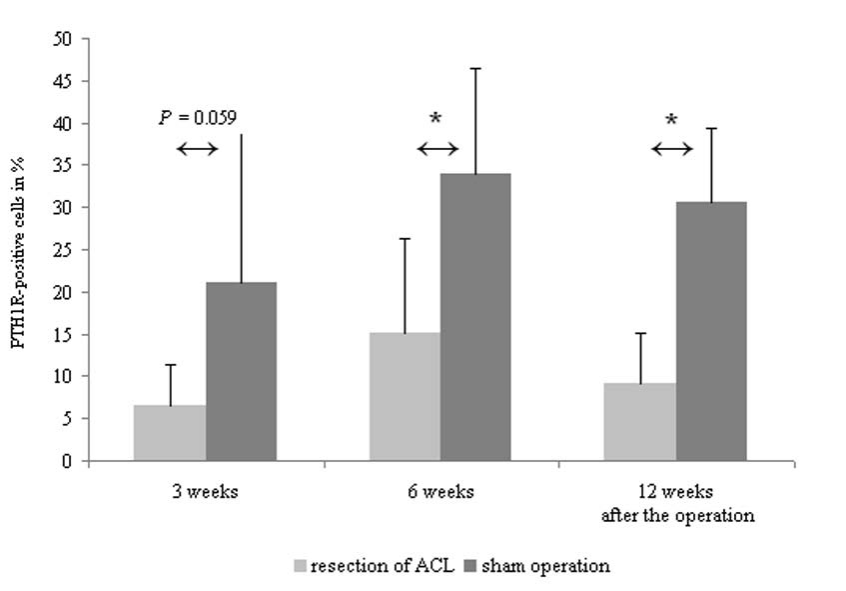
**Time course of the development of PTH1R expression in osteoarthritis**. The percentage of PTH1R+ chondrocytes was significantly increased in the anterior cruciate ligament (ACL) transection group after 6 weeks and 12 weeks. Values are the mean and SD.* = *P *< 0.05.

Immunohistochemical staining of PTH1R showed marked staining of the cell membrane in all cases. PTH1R was expressed mainly in the middle zones of cartilage. It was rarely found in the superficial zones (Figure 1). In group 1 (ACL resection), the percentage of PTH1R+ cells averaged 6.47% after 3 weeks, 15.14% after 6 weeks, and 9.15% after 12 weeks (Figure 2). In group 2 (sham operation), the percentage of PTH1R+ cells averaged 21.11% after 3 weeks, 33.90% after 6 weeks, and 30.53% after 12 weeks (Figure 2). In group 3 (control group), the percentage of PTH1R+ cells averaged 30.85%.

The percentages of PTH1R+ cells differed significantly between group 1 and 2 after 6 *(P *< 0.05) and 12 weeks *(P *< 0.01), but not after 3 weeks *(P *= 0.059). The percentages of PTH1R+ cells differed significantly between the ACL resected group and the control group at 3, 6 and 12 weeks respectively (*P *< 0.01).

**Figure 3:**
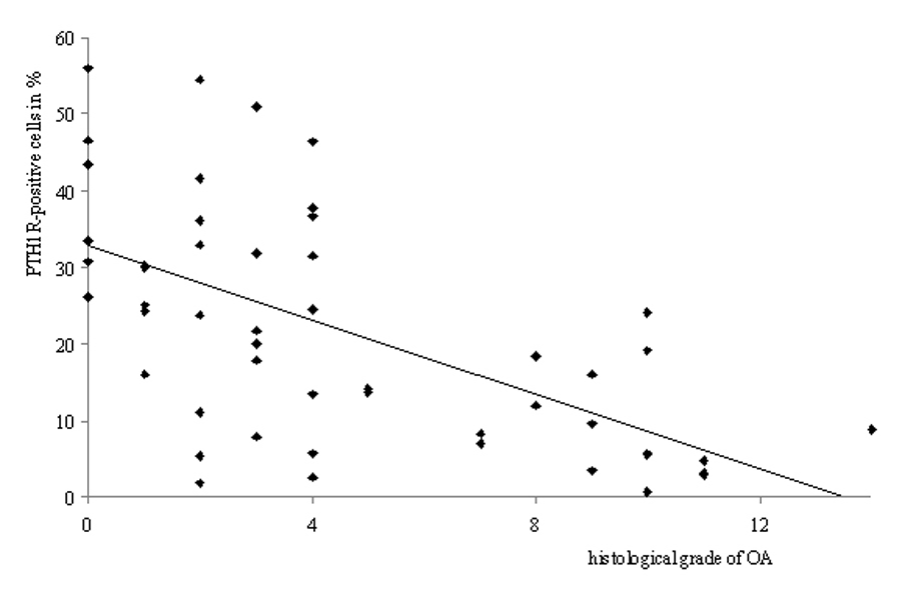
**Correlation between the histologic grade of osteoarthritis (OA) according to the Mankin scale and expression of PTH1R**. A negative correlation was demonstrated, with a Spearman's coefficient of -0.601 *(P *< 0.001).

**Figure 4:**
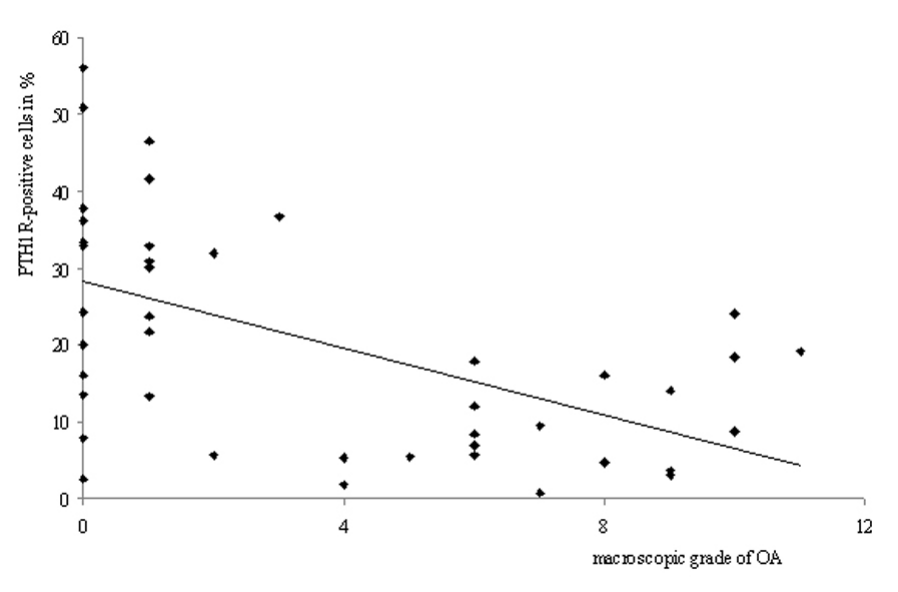
**Correlation between the macroscopic grade of osteoarthritis (OA) and the expression of PTH1R**. A negative correlation was demonstrated, with a Spearman's coefficient of -0.541 *(P *< 0.001).

There was a statistically significant negative correlation between the histologic grade of OA and the percentage of PTH1R+ chondrocytes (r_s _= -0.601, *P *< 0.001) (Figure 3). There was also a statistically significant negative correlation between the macroscopic grade of OA and the percentage of PTH1R+ chondrocytes (r_s _= -0.541, *P *< 0.001) (Figure 4).

## Discussion

The use of experimental animal models in which joint instability is induced through surgical intervention in an effort to clarify the mechanisms whereby the mechanical stress leads to OA development, have been widely used in the literature [18-21]. Whereas the mechanisms of the type 1 PTH/PTHrP receptor (PTH1R) in osteoblasts were addressed in several studies [11,22-25], the expression of the PTH1R and its correlation with macroscopic and histologic features over the time course in a model of experimentally induced early OA are largely unknown.

Our results confirm the expression of PTH1R in normal articular cartilage chondrocytes as detected by immunohistochemistry analysis [14]. We could demonstrate a negative linear correlation between PTH1R expression and macroscopic and histologic grades in the early course of OA.

This is consistent with findings that as disease severity in OA progresses, PTH1R expression and protein levels are reduced in human subchondral osteoblasts. Cases of severe, moderate, and mild OA patients who underwent total knee replacement surgery indicated a progressive decrease in PTH1R levels from -10% (mild) to -60% (severe) versus normal individuals [11]. Subchondral bone sclerosis is a major pathophysiological manifestation of OA, and it still is unknown if it precedes cartilage breakdown in OA. A study to determine the influence of osteoarthritic phenotype of subchondral osteoblasts on the phenotype of human chondrocytes demonstrated that sclerotic osteoblasts, but not nonsclerotic osteoblasts induced a significant decrease of PTH1R gene expression in chondrocytes [25]. In contrast, PTH1R gene expression was depressed in sclerotic osteoblasts [24]. Accordingly it was shown that proteinases like matrix metalloproteinase 13 (MMP-13) who have been proven to be the principal initiator of OA progression were significantly up-regulated in sclerotic osteoblasts compared with nonsclerotic osteoblasts [24]. MMP-13 are also highly expressed in the hypertrophic chondrocytes in response to joint instability [10]. Hypertrophic differentiation and chondrocyte apoptosis are known to be involved in OA development [26]. High levels of PTHrP have been found in the synovial fluid of osteoarthritic joints [13] and showed to regulate chondrogenesis in a manner that attenuates chondrocyte hypertrophy [7]. PTH participates in the regulation of cartilage growth and chondrocytic apoptosis [6]. Thus, it can be assumed that PTH1R participates in the underlying molecular mechanisms between cartilage degradation and subchondral bone remodelling and determines an important appearance in osteoarthritic progression.

Whereas the diffuse distribution of the stained cells in the middle zone in normal cartilage of our study was consistent with findings by other researchers [14], the distribution was not different in OA cartilage which is in contrast to findings in OA human cartilage at the time of joint replacement when receptor staining was relatively restricted to areas near the cartilage surface [14]. However, we confirmed that PTH1R is less expressed in OA cartilage with only a minority of cells expressing the receptor than in normal cartilage. It was shown in young adult bovine articular cartilage that chondrocytes of the radial zone occupied twice the volume and surface area of the chondrocytes of the superficial zone but were 10 times more synthetically active [27]. We hypothesize that PTH1R is down-regulated in the early course of OA due to the increased mechanical stimuli in the active radial and transitional zone and may be found in the superficial zone in late stages of OA when cartilage degradation is advanced.

In summary, the present study shows a decrease in the expression of the PTH1R receptor over the time course of OA. Further studies are needed to determine 1) the physiologic role of this receptor in normal articular cartilage, 2) whether PTH1R down-regulation is an underlying cause or a repair response in OA, 3) whether this pattern also applies to humans, and 4) whether new treatment approaches could evolve from this knowledge.

## Competing interests

In support of their research, none of the authors received grants or outside funding. None of the authors received payments or other benefits or a commitment or agreement to provide such benefits from a commercial entity.

## Authors' contributions

All authors read and approved the final manuscript. CB drafted the manuscript and participated in data and statistical analysis. TS participated in the conception of the study, participated in the specimen preparation and was responsible for immunohistochemical staining. PR participated in the surgical procedures, specimen preparation and macroscopic and histological grading of OA. He carried out the quantitative counting of cells and participated in immunohistochemical staining. SO participated in the statistical analysis and manuscript preparation. AS participated in the surgical procedures, specimen preparation and macroscopic and histological grading of OA. SFW participated in the conception of the study and supervised the protocol. COT was responsible for the initial conception of the research question, supervising the protocol and manuscript preparation.
